# Physicochemical stability of corn protein hydrolysate/tannic acid complex‐based β‐carotene nanoemulsion delivery system

**DOI:** 10.1002/fsn3.4160

**Published:** 2024-04-18

**Authors:** Yong‐Hui Wang, Sheng‐Hua He, Ji‐Hong Huang, Wei‐Yun Guo, Xue‐Li Gao, Guang‐Hui Li

**Affiliations:** ^1^ Food and Pharmacy College Xuchang University Xuchang People's Republic of China; ^2^ Collaborative Innovation Center of Functional Food Green Manufacturing Xuchang People's Republic of China

**Keywords:** corn protein hydrolysate, nanoemulsion, physicochemical stability, tannic acid, β‐carotene

## Abstract

Moderate non‐covalent interaction of protein and polyphenols can improve the emulsifying property of protein itself. The corn protein hydrolysate (CPH) and tannic acid (TA) complex was successfully used to construct nanoemulsion for algal oil delivery. There has been no study on the feasibility of this nanoemulsion delivery system for other food functional components, for example, β‐carotene (β‐CE). CPH/TA complex‐based nanoemulsion system for β‐CE delivery was studied, focusing on the effect of β‐CE content on the physicochemical stability of the nanoemulsions. The nanoemulsion delivery systems (dia. 150 nm) with low viscosity and good liquidity were easily fabricated by two‐step emulsification. The nanoemulsions with high β‐CE content (>71.5 μg/mL) significantly increased (*p* < .05) the emulsion droplet size. However, there was no significant (*p* > .05) effect of β‐CE content on polydispersity index (PDI) and zeta potential of the nanoemulsions. The storage (30 days) experiment results demonstrated that the droplet size of the nanoemulsions with varying β‐CE content increased slightly during storage. However, the PDI values showed a slightly decreasing trend. Zeta potentials of the nanoemulsions showed no noticeable change during storage. Moreover, after storage of 30 days, the retention ratios of β‐CE were found to be up to 90%, which suggests an excellent protective effect for β‐CE by the nanoemulsion systems. The CPH/TA complex stabilized nanoemulsions could aggregate in gastric condition, but the β‐CE content did not have obvious effect on the digestive stability of the nanoemulsions. The CPH/TA complex could be employed as an emulsifier to construct a physicochemical stable nanoemulsion delivery system for lipophilic active components.

## Graphical abstract

β‐Carotene (β‐CE) nanoemulsion can be fabricated by the complex of corn protein hydrolysate (CPH) and tannic acid (TA); CPH–TA complex stabilized β‐CE nanoemulsion showed long‐term physicochemical stability. With an increase in β‐CE content, the droplet size of the nanoemulsion tends to increase.

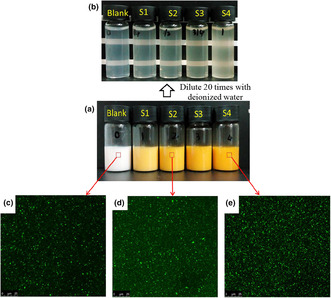

## 1 INTRODUCTION

There is an increasing trend of consuming foods that provide health benefits and treat certain diseases, such as foods with added proteins, vitamins, minerals, and antioxidants. However, poor water solubility makes bioactive compounds challenging to incorporate into the food matrix. The emulsion‐based delivery system has been extensively studied for encapsulating the lipophilic bioactive components in pharmaceuticals. Nanoemulsions and conventional emulsions of oil in water are both thermodynamically unstable systems with lipid droplets coated with emulsifiers dispersed in aqueous media (Ahmed et al., 2012). The nanoemulsions are kinetically metastable systems with either a transparent or a translucent appearance, and their mean droplet radius is <100 nanometers (Choi & McClements, 2020; Mehmood et al., 2018). Because of the smaller droplet size, nanoemulsions generally exhibit higher stability, solubility, and bioavailability than conventional emulsions (Acosta, 2009; McClements & Rao, 2011; Tadros et al., 2004). It is possible to manufacture the nanoemulsions using technologies already widely used by the food industry, such as sonication and microfluidization, which are economically viable (Chen et al., 2020; Mason et al., 2006).

β‐Carotene (β‐CE) is an essential source of provitamin A and a natural edible pigment. Due to its long, highly unsaturated conjugated hydrocarbon chain, β‐CE can effectively quench singlet oxygen and free radicals, thus protecting foods and biological tissues from oxidation (Monego et al., 2017). However, the double bonds in β‐CE also make it highly prone to oxidation, leading to color fading and loss of biological activity, thereby reducing its efficacy (Yi et al., 2018). Therefore, the application of β‐CE in foods is affected due to its instability and low oral bioavailability. Emulsion‐based delivery systems, like nanoemulsion, are particularly suitable for delivering lipophilic bioactive components (e.g., carotenoids) because the encapsulation processes can protect the components from oxidation (Chaari et al., 2018).

Incorporating phenolic compounds (e.g., tannic acid) into proteins effectively improves their emulsifying and antioxidant properties (Wang et al., 2016). Research has shown that (tea) polyphenols could improve the colloidal stability and increase the retention rate of β‐CE during storage of the nanoemulsions (Meng et al., 2019). It also demonstrated that chitosan–gallic acid conjugates could improve the physicochemical stability of β‐CE nanoemulsion (Yi et al., 2021). Moreover, β‐CE physicochemical stability and bioaccessibility could also be improved in tea polysaccharide conjugates stabilized nanoemulsions (Li et al., 2021).

Recently, plant‐derived proteins have attracted more attention in the food industry because of their advantages in food safety risks, sustainability, and health effects (Nasrabadi et al., 2021). Corn gluten meal (CGM), a byproduct of corn starch processing, contains protein as high as 60%. However, due to its low water solubility and unbalanced amino acid composition, the CGM protein is mainly used as animal feed (Hu et al., 2020). Enzymatic hydrolysis of CGM not only hugely improves its water solubility but also produces plenty of corn protein hydrolysate (CPH) with prominent physiological functions, for example, antioxidant activity and antiangiotensin‐converting enzyme (ACE) inhibitory activity (Shen et al., 2020; Wang et al., 2020). However, the emulsification property of CPH is low due to its high hydrophilicity, which is especially true for CPH with a high degree of hydrolysis. Research shows that the interaction between CPH and tannic acid (TA) resulted in the complex exhibiting better emulsifying properties than pure CPH (Wang et al., 2019). We have previously confirmed that the non‐covalent complexes of corn protein (zein) hydrolysate and tannic acid could serve as an effective emulsifier in the nanoemulsion delivery system (Wang et al., 2016). However, the feasibility of the CPH/TA complex‐based nanoemulsion delivery system for other food functional components (e.g., β‐CE) and the effect of β‐CE content on the physicochemical stability of the nanoemulsion system have not been studied yet. Thus, in this study, we present CPH/TA complex‐based nanoemulsions for β‐CE delivery, focusing on the effect of β‐CE content on the physicochemical stability of the nanoemulsions.

## 2 MATERIALS AND METHODS

### 2.1 Materials

Corn gluten meal (CGM) (protein content 52.9%) was obtained from Zhucheng XingMao Corn Development Co., Ltd. (Zhucheng, China). Corn protein hydrolysate (CPH) was prepared from CGM according to our previous report (Shen et al., 2020; Wang et al., 2020). The degree of hydrolysis of CPH was 25%, determined by the pH‐Stat method. β‐CE (purity ≥95%) was purchased from Sigma‐Aldrich (St. Louis, MO, USA), tannic acid (≥98%) and Nile red (≥95%) from Aladdin Biochemical Technology (Shanghai, China), and corn oil from the local supermarket. Pepsin (15,000 μ/g), bile extract (≥60%), and pancreatin (4000 μ/g) were purchased from Yuanye Biotechnology Co., Ltd. (Shanghai, China). Other chemicals used in this study were of analytical grade.

### 2.2 Preparation of the CPH/TA complex

Stock solutions of CPH (20 mg/mL) and TA (20 mg/mL) in phosphate buffer solution (pH 7.0, 10 mM) were prepared under room conditions. Before the preparation of CPH/TA complex solution, CPH and TA solutions were further diluted by the phosphate buffer solution, and the final concentrations of CPH and TA in the complex solution were 10 and 3 mg/mL, respectively. As a previous study showed, CPH and TA should be mixed at a mass ratio of 10:3, a proportion at which the complex solutions exhibit the best emulsification properties (Wang et al., 2016).

### 2.3 Preparation of β‐CE nanoemulsions

Corn oil was heated to 50°C and β‐CE (0.5%, w/v) was dissolved in the heated corn oil by stirring with a magnetic stirrer at 100 rpm (revolutions per minute) for 10 min. After standing for 1 h at room temperature, the β‐CE solution was centrifuged at 5000× *g* for 10 min at 20°C to remove the trace amount of sediment. Using 15 mL vials, 0, 0.1, 0.2, 0.3, and 0.5 g of the β‐CE solutions was added into the CPH/TA solution (9.5 mL), respectively, and additional corn oil (without β‐CE) was added to acquire the same mass (0.5 g) of the total oil phase. The samples were named Blank, S1, S2, S3, and S4, respectively. Emulsification was performed first using a shearing mixer (T10 Ultra‐Turrax, IKA, Staufen, Germany) at 5000 rpm for 2 min. Then, the obtained coarse emulsions were further treated with ultrasonic equipment (Omni International, Kennesaw, GA, USA) for 5 min at 70% output power. This emulsification processing was performed on an ice bath. Sodium azide (0.04 wt%) was added to the vials to prevent microbial growth. The vials were tightly sealed and stored at ambient conditions for 30 days.

### 2.4 Determination of the β‐CE content and oil encapsulation efficiency

The β‐CE content of the emulsions was determined according to the spectrophotometric method (Mehmood et al., 2021). Briefly, the samples (1 mL) were extracted from the mixture of n‐hexane (3 mL) and ethanol (3 mL). After mixing using a vibrator, the hexane phase was separated by centrifuge and repeated twice for extraction. The combined hexane phases were then further diluted to 10 mL with n‐hexane, and the absorbance was measured at 450 nm using a ultraviolet (UV) spectrophotometer. The β‐CE content of the nanoemulsions was computed through a predetermined calibration curve (*y* = 5.807*x* − 0.087, *R* ^2^ = 0.9991).

The encapsulation efficiency of the oil phase was determined by the method of Yang et al. (2014). The unburied oil phase was extracted as follows: adding 10 mL hexane into the β‐CE nanoemulsion, shaking vigorously with a vortex mixer for 1 min. The mixture was centrifuged at 5000× *g* for 5 min at 20°C, and the supernatant (8 mL) transferred into a weighted tube. Evaporation was conducted under nitrogen to remove the hexane. The obtained oil phase was weighted to the nearest 0.1 mg. The encapsulation efficiency was computed using the formula given below:

$$\text{Encapsulation efficiency}\;\left(\%\right)=\frac{W_{0}-W_{1}}{W_{0}}\times 100.$$

Here, *W*_0_ and *W*_1_ represent the initial total oil weight and the unburied oil weight, respectively.

### 2.5 Confocal laser scanning microscope (CLSM)

To directly observe the oil distribution of the emulsions, images of the freshly prepared emulsions were recorded with a Leica TCS SP5 CLSM (Leica Microsystems Inc., Heidelberg, Germany). The entire protocol can be found in the previously published report (Wang et al., 2016).

### 2.6 Colloidal characterization

To evaluate the physical properties and stability of the emulsions, mean particle diameter (*z*‐average), polydispersity index (PDI), and zeta potential were measured by a dynamic light scattering and micro‐electrophoresis device (Malvern Instruments Co. Ltd., Worcestershire, UK). Before analysis, the emulsion samples were diluted 100 times with deionized water to avoid multiple scattering effects. The experiment was performed under ambient conditions.

### 2.7 Storage stability of β‐CE nanoemulsion

The samples were taken out and analyzed at varying intervals during storage. The retention percentage expressed the chemical stability of the β‐CE nanoemulsion. Retention percentage (%) = 100 × *C*_*t*_/*C*_0_, where *C*_*t*_ is the content of β‐CE after storage for *t* days and *C*_0_ is the initial β‐CE content before storage. The changing of *z*‐average droplet size during the storage represents the physical stability of the nanoemulsions.

### 2.8 In vitro digestion of β‐CE nanoemulsion

An in vitro gastrointestinal tract (GIT) model, consisting of stomach and intestine stages, was used to simulate the biological fate of ingested samples. The emulsions were passed through a GIT, and samples were taken out after each stage. The process was carried out by the method of Zhou et al. (2018), with slight modifications. Briefly, 10 mL of the initial sample was diluted two times by distilled water and 10 mL of gastric juice (3.5 mg/mL pepsin and 0.15 M sodium chloride (NaCl), pH 2.0) was added. The mixture was incubated at 37°C for 1 h with continuous agitation at 100 rpm. After gastric digestion, the digesta was immediately adjusted to pH 7.0 using NaOH. Simulated intestinal juice (15 mL, containing 1.0 mg/mL pancreatin, 20.0 mg/mL bile extract, and 10 mmol/L calcium chloride (CaCl_2_)) was added and incubated at 37°C for 2 h, with the pH maintained at 7.0 by adding 0.25 M NaOH drop by drop.

### 2.9 Statistical analysis

All experiments were conducted at least in triplicate independently. Data were compared using the analysis of variance (ANOVA) method by SPSS software, version 19.0 (SPSS Inc., Chicago, IL, USA). The differences between the mean values of the data were compared at a significant level of 5%.

## 3 RESULTS AND DISCUSSION

### 3.1 Appearance and microstructure of β‐CE nanoemulsions

**FIGURE 1:**
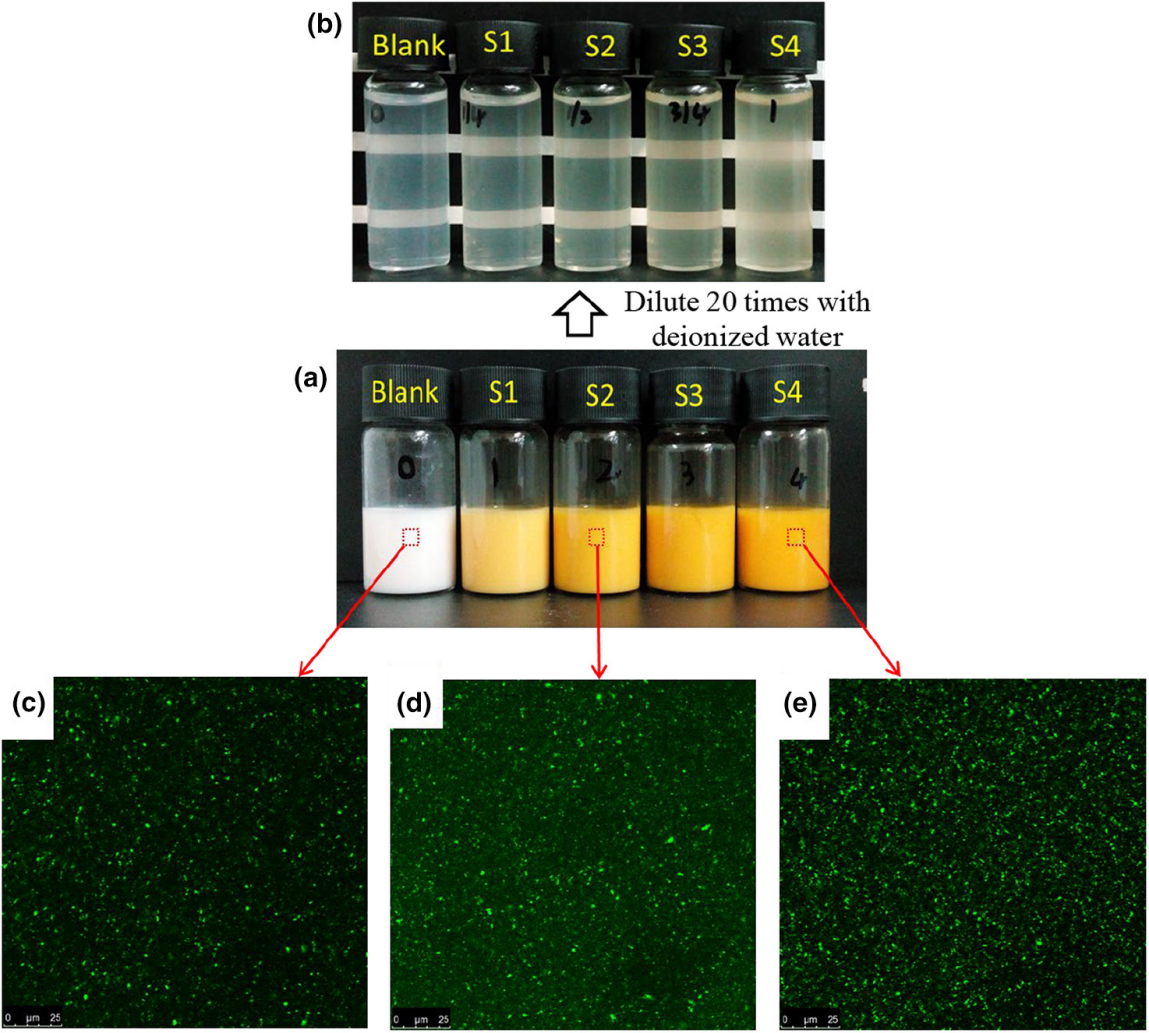
Visual appearance (a, b) and CLSM images of β‐CE nanoemulsions (c–e). Blank was the emulsion without β‐CE addition, and S1–S4 were the emulsions with β‐CE content of 35.1, 71.3, 100.5, and 142.2 μg/mL, respectively.

After preparation, all the emulsions had visual stability without phase separation. With increasing β‐CE proportions, the yellow color of the emulsion was gradually intense (Figure 1a). Moreover, all the emulsions remained visually stable after being diluted 20 times with deionized water (Figure 1b). The transparent or translucent appearance of the diluted emulsions is displayed in accordance with typical optical properties of nanoemulsions (Wooster et al., 2008). All samples had very low viscosity, good liquidity, and exhibited non‐Newtonian fluid characteristics, with the shear rate increasing, samples’ apparent viscosity steadily reducing (Figure S1).

Confocal laser scanning microscopy (CLSM) images of the emulsions showed that the CPH/TA complex stabilized oil droplets are all submicron in size and are nearly close to the detection limit of CLSM (Figure 1c–e). These results showed that all emulsions are similar in size, indicating that the β‐CE content of the oil phase had no pronounced effect on the droplet size. Additionally, the nanoemulsions showed excellent stability against droplet coalescence after storage of 30 days under room conditions (Figure S2).

### 3.2 β‐CE content and encapsulation efficiency of oil phase

**FIGURE 2:**
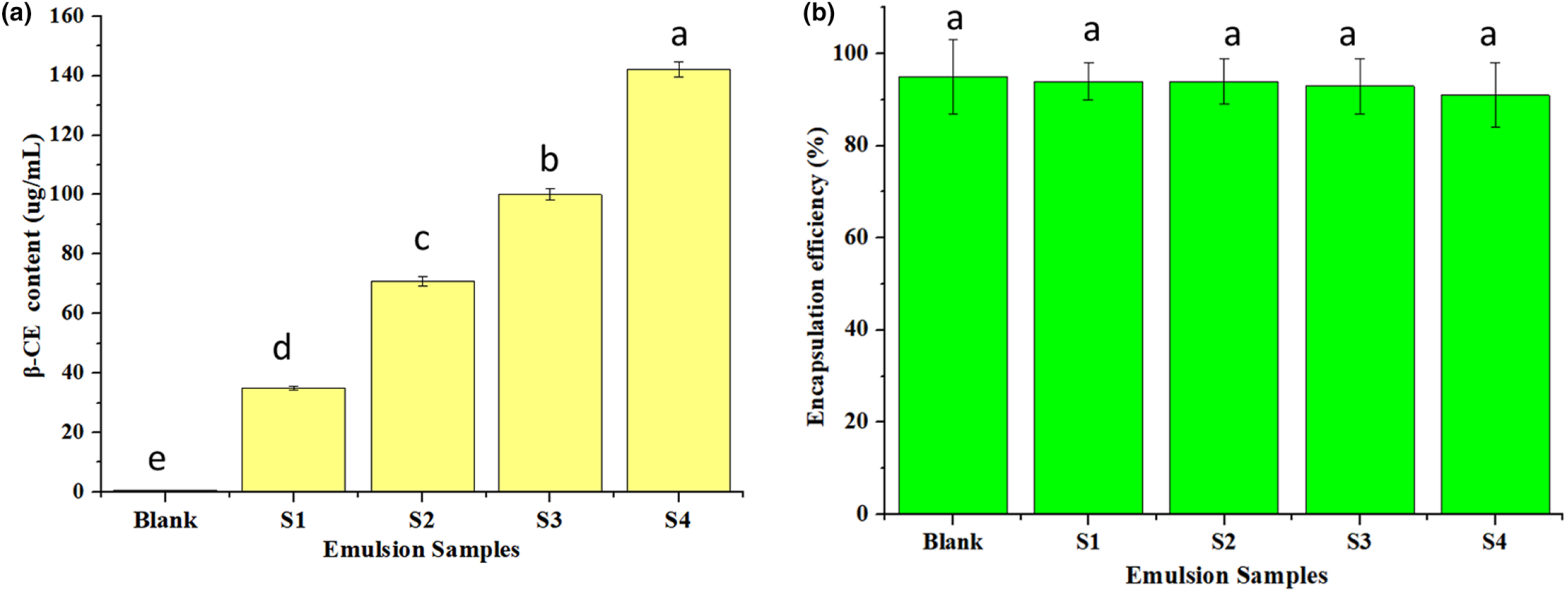
β‐Carotene (β‐CE) contents of the freshly prepared nanoemulsions (a) and encapsulation efficiency of the nanoemulsions (b). The different letters above the bars mean significant differences between the groups (*p* < .05). Blank was the emulsion without β‐CE addition, and S1–S4 were the emulsions with β‐CE content of 35.1, 71.3, 100.5, and 142.2 μg/mL, respectively.

β‐Carotene content of the nanoemulsions, labeled S1, S2, S3, and S4, is shown in Figure 2a. As expected, due to the low content of β‐CE in the refined edible corn oil, almost no β‐CE was detected in the control sample (Blank). For the samples of S1 to S4, the β‐CE concentration increased from 35.1 to 142.2 μg/mL. Additionally, the encapsulation efficiency of the oil phase for all nanoemulsions was above 95%. No significant difference (*p* > .05) in the encapsulation efficiency was observed for the nanoemulsions with varying β‐CE content (Figure 2b). The results indicated that the content of lipophilic bioactive agents in the oil phase did not affect the encapsulation efficiency under these test conditions.

### 3.3 Colloidal properties of the emulsions

**FIGURE 3:**
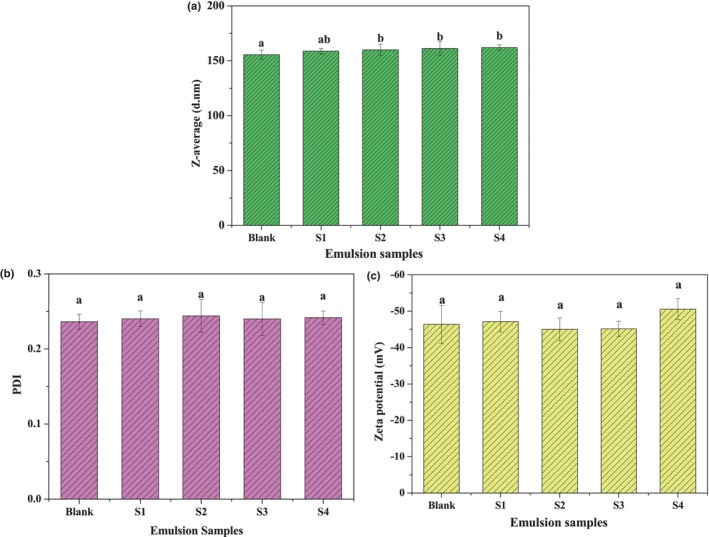
The *z*‐average size (a), PDI (b), and zeta potential (c) of the freshly prepared nanoemulsions. The different letters above the bars mean significant differences between the groups (*p* < .05). Blank was the emulsion without β‐CE addition, and S1–S4 were the emulsions with β‐CE content of 35.1, 71.3, 100.5, and 142.2 μg/mL, respectively.

The colloidal properties of the emulsions were readily characterized by z‐average, PDI, and zeta potential, which are important indicators for the physical stability of the emulsions. The particle size over time is an important parameter to evaluate the physical stability of the nanoparticle during storage (Chaari et al., 2018). Generally, small size, low PDI, and high zeta potential imply that the particles have high physical stability. Figure 3a shows that no significant changes (*p* > .05) occurred in the particle size of the nanoemulsions with varying β‐CE content (from S1 to S4). However, compared with the blank emulsion (155.5 nm), the particle size of the samples with high β‐CE content increased significantly (*p* < .05), for example, the size of S4 increased to 162 nm. Generally, the droplet size distribution of an emulsion could be influenced by modulating the emulsifying parameters, for example, intensity, duration, and system composition (type and amount of oil and emulsifier) (Choi & McClements, 2020). This result suggested that lipophilic nutraceutical content also affects the droplet size of the emulsion on the premise that the emulsions have the same oil phase volume fraction. The reason may be explained by the rise of oil phase viscosity. This hypothesis is supported by a previous study in which nanoemulsions prepared with high‐viscosity oils, such as long‐chain triglycerides (LCT), were considerably larger in size than those with low‐viscosity oils such as hexadecane (Wooster et al., 2008).

The results from the PDI (Figure 3b) show that the emulsions with and without β‐CE encapsulation had no significant differences (*p* > .05). The PDI values were between 0.2 and 0.25, meaning that the emulsions stabilized by CPH/TA complexes have low polydispersity. Zeta potential measurements indicated that the droplets of the emulsions have negative charge (Figure 3c), mainly caused by the ionization of anionic groups (partial amino acid residues and free fatty acids). The high negative zeta potential values of the emulsions, between −40 and −50 mV, indicate that sizeable electrostatic repulsion exists between the emulsion droplets. This result is similar to our previous report, in which zein hydrolysate–tannic acid complex stabilized algal oil nanoemulsions exhibited similar colloidal properties (Wang et al., 2016).

### 3.4 Physical stability during storage

**FIGURE 4:**
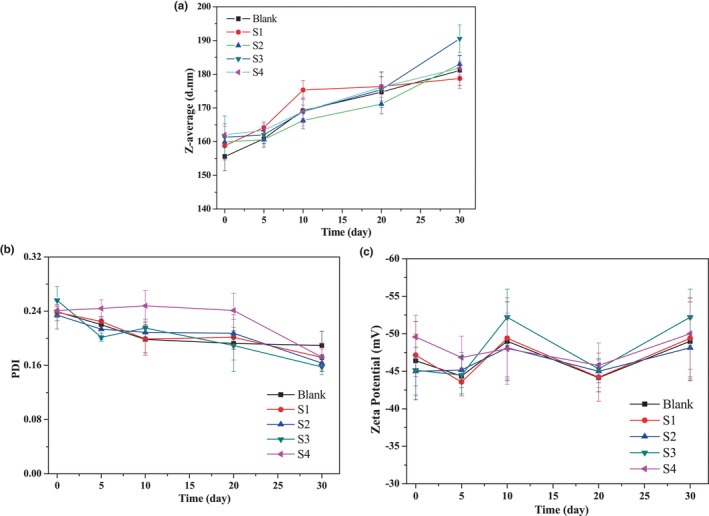
The *z*‐average size (a), PDI (b), and zeta potential (c) of the nanoemulsions during storage. Blank was the emulsion without β‐CE addition, and S1–S4 were the emulsions with β‐CE content of 35.1, 71.3, 100.5, and 142.2 μg/mL, respectively.

During the storage of the β‐CE nanoemulsions, the *z*‐average droplet size, PDI, and zeta potential at different times are shown in Figure 4. With increasing storage time, *z*‐average droplet size of all the nanoemulsion samples showed a slightly increasing trend (Figure 4a). The *z*‐average droplet size of all nanoemulsions was increased by approximately 20 nm after 30 days of storage. This observation suggested that β‐CE content had no effect on the stability of the emulsion, but storage duration did. Generally, the PDI of emulsion will usually increase due to the process of the Ostwald ripening. This process caused the smaller droplet size to shrink and the larger droplet size to grow because of the diffusion of molecules from the dispersed phase to the continuous phase and the difference in molecular curvature (Siraj et al., 2021). However, an interesting observation can be found in Figure 4b. For all investigated emulsions, the PDI shows a steadily decreasing trend, as opposed to the expected increasing trend. This result may be attributed to the coalescence of smaller droplets, a process in which the intermolecular attractions and surface tension result in dispersed phase collision, merging, and forming larger droplets.

Additionally, the zeta potential of all the nanoemulsions showed no noticeable change during the storage period of 30 days. The absolute values were maintained above 45 mV (Figure 4c). Repulsive forces between colloids, because of excluded volume, charges of the particle surfaces, or ‘steric’ interactions derived from brush‐like coatings of polymer surfaces, can effectively prevent particles from aggregating; finally, the colloids will remain homogeneous in the long term (Mason et al., 2006). These results suggest that the CPH/TA complex has an excellent emulsifying ability, which can maintain the stability of the oil–water (O/W) interface over a long period.

### 3.5 Chemical stability during storage

**FIGURE 5:**
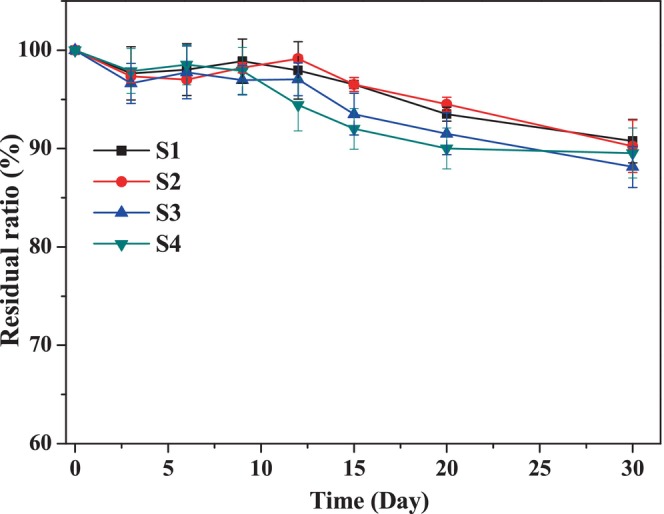
β‐Carotene (β‐CE) retention ratio of the emulsion during storage at ambient condition. Blank was the emulsion without β‐CE addition, and S1–S4 were the emulsions with β‐CE content of 35.1, 71.3, 100.5, and 142.2 μg/mL, respectively.

During storage, the stability of encapsulated active substances, such as β‐CE, is essential for ensuring the particle's ability to function as a delivery system. Due to its strong lipophilicity, β‐CE tends to be confined to the oil phase of the emulsion system. van der Waals interactions could regulate the binding effect of β‐CE and the fatty acid chain (Borba et al., 2019). β‐CE and lipids have similar oxidation mechanisms when exposed to heat, light, and oxygen during storage, but β‐CE can suffer from oxidation and isomerization. Encapsulation can potentially protect active substances from deteriorating conditions, helping to maintain long‐term stability (Hong et al., 2017). Figure 5 shows the β‐CE retention ratio of the emulsion during 30 days of storage. The β‐CE retention ratios did not significantly change until 15 days of storage, after which the β‐CE retention ratios significantly lowered. After 30 days of storage, the retention ratios were still above 90%. This result is quite different from the previous report (Borba et al., 2019), in which lower β‐CE content of the formulation caused higher β‐CE retention rates. This phenomenon may be explained by the physical stability of the emulsions and the chemical properties of TA.

The improved chemical stability of β‐CE nanoemulsions stabilized by the CPH/TA complex can be attributed to the increased emulsifying ability of CPH itself after the complexation. Due to high emulsifying properties, the CPH/TA complex readily concentrates at the oil–water (O/W) interface, which resulted in the nanoemulsions having high oil encapsulation efficiencies and good physical stability. Moreover, the TA has excellent antioxidative abilities, contributing to the chemical stability of β‐CE. Reports showed that the tannic acid has practical scavenging activities for 2,2‐diphenyl‐1‐picrylhydrazyl (DPPH), 2,2′‐azinobis(3‐ethylbenzothiazoline‐6‐sulfonic acid) radical cation (ABTS+), superoxide anion, hydrogen peroxide (H_2_O_2_), and low concentration of TA (15 μg/mL) could inhibit 97.7% lipid peroxidation for the linoleic acid emulsion (Gülçin et al., 2010). Li et al. (2019) found that TA addition could effectively inhibit temperature‐induced β‐CE degradation for the emulsions stabilized by two plant‐based emulsifiers (quillaja saponin and gum arabic). Therefore, the phenolic groups of TA contributed to the excellent oxidative stability of the CPH/TA complex. Additionally, CPH exhibits antioxidant properties, which may increase the chemical stability of the encapsulated β‐CE. It has been reported that corn gluten meal hydrolysates could inhibit lipid deterioration by reducing the formation of hydroperoxides and thiobarbituric acid reactive substances (TBARS), and it could serve as a potential antioxidant for food applications (Shen et al., 2020). The impressive antioxidant properties of CPH have also been reported in the relevant studies (Hu et al., 2020; Liang et al., 2017).

### 3.6 Physical stability of β‐CE nanoemulsion in a digestion model

**FIGURE 6:**
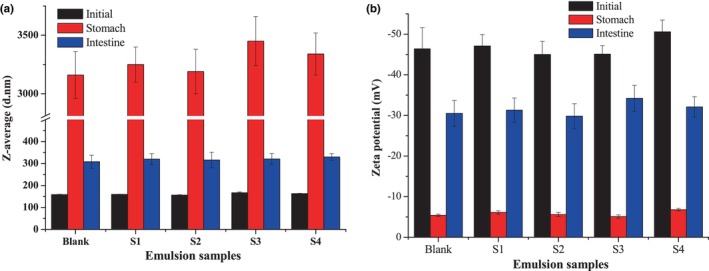
The *z*‐average droplet size (a) and zeta potential (b) of the nanoemulsions with varying β‐CE content in the two digestion stages. Blank was the emulsion without β‐CE addition, and S1–S4 were the emulsions with β‐CE content of 35.1, 71.3, 100.5, and 142.2 μg/mL, respectively.

The z‐average droplet size of the CPH/TA complex‐based nanoemulsions is shown in Figure 6a. All the β‐CE nanoemulsions became highly aggregated in gastric condition, especially in stomach stage, with the size of the aggregates increased to several micrometers. This phenomenon could be attributed to the low pH value and high ionic strength. The reduction in pH and increase in ionic strength in the simulated stomach altered electrostatic interactions between droplets. It could be well demonstrated according to the reduction in magnitude of the zeta potential of the droplets in the gastric phase (Figure 6b). Usually for protein‐based emulsions, the role of pepsin is also an important factor for the destabilization of emulsions (Zhou et al., 2018). The hydrolysis of the proteins adsorbed at the oil–droplet interface can also cause the aggregation of emulsions (Zhang et al., 2017). In this case, however, the role of pepsin may be almost negligible due to CPH, especially CPH/TA complex being insensitive to pepsin. Moreover, it can be found that β‐CE content in the oil phase did not have an obvious effect on the digestion stability.

## 4 CONCLUSION

The CPH/TA complex‐based β‐CE nanoemulsion delivery system with low viscosity and good liquidity could be easily fabricated. With an increase in β‐CE content, the droplet size of CPH/TA complex stabilized nanoemulsions tends to increase, especially true for the emulsions with high β‐CE content (>71.5 μg/mL). There is no significant (*p* > .05) effect of β‐CE content on the PDI and zeta potential of the nanoemulsions. The results from storage at ambient conditions showed that the droplet size of the nanoemulsions increased during storage, and the average increase in amplitude was approximately 20 nm. The PDI values decreased slightly during storage, but the zeta potentials of the nanoemulsions showed no noticeable change. Moreover, the β‐CE content of the nanoemulsions has no apparent effect on the chemical stability of β‐CE itself during storage. After 30 days of storage, the retention ratios of β‐CE were still above 90%, showing that the nanoemulsion delivery system provides an excellent protective effect on the chemical stability of β‐CE. The CPH/TA complex‐based nanoemulsions exhibited destabilization phenomenon in gastric condition, but the β‐CE content in oil phase did not have obvious effect on the digestive stability of the nanoemulsions. This study suggests that the CPH/TA complex can be used as an effective emulsifier to construct nanoemulsion delivery systems for lipophilic active components.

## AUTHOR CONTRIBUTIONS

**Yong‐Hui Wang**: Conceptualization, Methodology, Data curation, Formal analysis, Writing‐original draft. **Sheng‐Hua He**: Supervision, Writing‐review & editing, Assisting in the completion of morphology detection. **Ji‐Hong Huang**: Resources, Visualization, Supervision. **Wei‐Yun Guo**: Data curation, Investigation, Supervision. **Xue‐Li Gao**: Project administration, Writing‐reviewing and editing, Funding acquisition. **Guang‐Hui Li**: Methodology, review and editing.

## CONFLICT OF INTEREST STATEMENT

The authors declare no conflicts of interest.

## Supporting information


**Fig. S1.**.

## Data Availability

Data available on request from the authors.
