# Supplementary material for: Physicochemical stability of corn protein hydrolysate/tannic acid complex‐based β‐carotene nanoemulsion delivery system
Source: Food Sci Nutr. 2024 Apr 18;12(7):5111–20. doi: 10.1002/fsn3.4160 (PMC11266910; doi:10.1002/fsn3.4160)
Supplement: Supplementary file 1 — Fig. S1.. [file FSN3-12-5111-s001.docx]

**Electronic Supplementary Information (ESI)**

**Physicochemical stability of** **corn protein hydrolysate-tannic acid complex based β-carotene nanoemulsion delivery system**

Yong-Hui Wang, Sheng-Hua He, Ji-Hong Huang, Wei-Yun Guo, Xue-Li Gao, Guang-Hui Li

Food and Pharmacy college, Xuchang University, Xuchang 461000, People’s Republic of China；

Collaborative Innovation Center of Functional Food Green Manufacturing, Xuchang, 461000, Henan Province, People’s Republic of China

Corresponding author: Yong-Hui Wang; Ji-Hong Huang

1. mail address:yonghuione@126.com; [huangjih1216@126.com](mailto:huangjih1216@126.com)


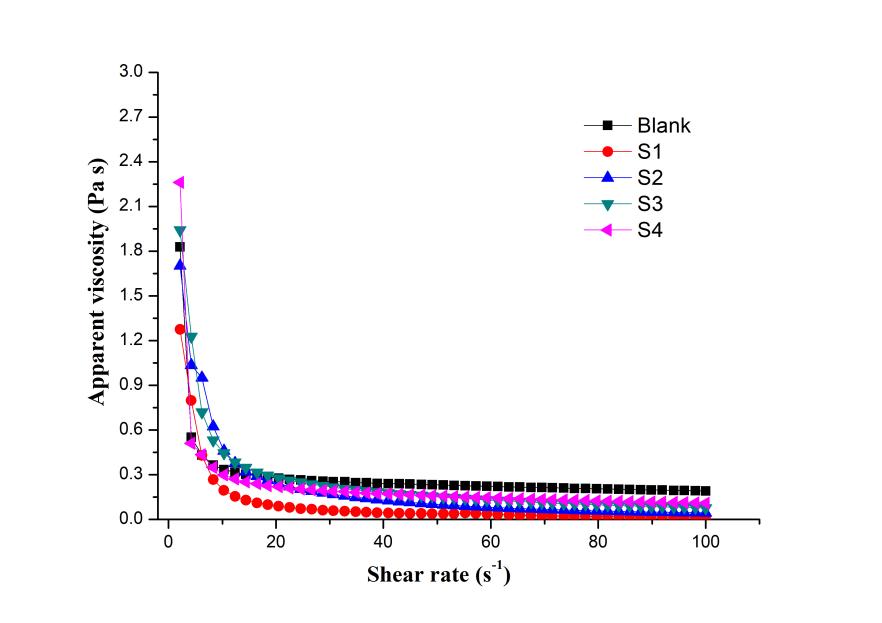


**Fig. S1.** Apparent viscosity of β-carotene nanoemulsiones stabilized by corn protein hydrolysate-tannic acid complex with different β-CE content. Blank was the emulsion without β-CE addition, and S1-S4 were the emulsions with β-CE content of 35.1, 71.3, 100.5 and 142.2 μg/mL, respectively.

**Fig. S2.** Appearance of the nanoemulsiones after 30 days storage at ambient condition. Blank was the emulsion without β-CE addition, and S1-S4 were the emulsions with β-CE content of 35.1, 71.3, 100.5 and 142.2 μg/mL, respectively.
